# Characterization and properties of manganese oxide film coated clinoptilolite as filter material in fixed-bed columns for removal of Mn(II) from aqueous solution

**DOI:** 10.1038/s41598-023-44611-8

**Published:** 2023-10-14

**Authors:** Xing Jin, Jinxiang Fu, Pengfei Yu, Di Luo

**Affiliations:** School of Municipal and Environmental Engineering, Shenyang Jianzhu University, Shenyang, 110000 China

**Keywords:** Pollution remediation, Composites

## Abstract

A new filter material, manganese oxide film coated clinoptilolite (MOFCC), was characterized and introduced to explore the effect in treating high concentration of manganese (1.71–2.12 mg L^−1^) from aqueous solution in fixed-bed column. Adsorption behavior of Mn(II) can be approximately described with the Langmuir isotherm. During the continuous 30 days filtration experiment, the removal rate of Mn(II) has maintained to be above 95.51%, the accumulated removal amount (806.42 mg) is much higher than the theoretical adsorption capacity (89.71 mg), which indicated that the removal of manganese by MOFCC includes both adsorption and auto-catalytic oxidation process, and it does not require a start-up period. SEM, EDS, XPS, XRD, ZETA potential and BET analyses were used to observe the surface properties of MOFCC. The manganese oxide film of MOFCC exhibits in clusters, apparently on occupied surface, the main component of the manganese oxide film is (Na_0.7_Ca_0.3_)Mn_7_O_14_·2.8H_2_O, the specific surface area of MOFCC is 38.76 m^2^ g^−1^, and the pore size is concentrated in the range of 3–40 nm, within the mesoporous range mesopores. pH_pzc_ (point of zero charge) value is about 2.36. The characteristics of MOFCC make it an excellent manganese removal filter material for water treatment plant. Therefore, there is a long-term practical significance to develop new system for deep removal of manganese based on MOFCC.

## Introduction

Manganese is one of the essential elements in the human body, but excessive intake of manganese can cause a decline in intelligence, vision, memory, and motor function^1,2^. Groundwater is a widely used source of drinking water, affected by conditions such as manganese minerals in soil, climate, and hydrological environment, manganese content in groundwater may exceed the standard^3,4^. Therefore, manganese removal from groundwater has received wide attention. Limited by economic factors, secondary pollution, complex operations, and other factors, membrane filtration^5^, ion exchange^6^, adsorption^7^ and other methods are difficult to widely used in water treatment plant. The rapid sand filtration (RSF)^8^ method is a highly feasible method, but the start-up period often takes up to 3–6 months^9^, greatly affecting the widespread use of RSF. Besides, the manganese removal mechanism of RSF is still unclear. Physicochemical^10^ and biological^11^ oxidation pathways were difficult to separate in RSF, and there is also a certain synergistic effect between the two oxidation methods^12^. Although some manganese oxidizing bacteria species could accelerate the start-up of the sand filter^13^, it still took 2–3 months to colonize, stabilize and accumulate^14^. Therefore, accelerating the start-up period through physicochemical oxidation is the focus of RSF method of manganese removal.

The research on the catalytic oxidation process of Mn(II) on the surface of metal oxides has been carried out for a long time, montmorillonite, kaolinite, goethite^15^ and γ-FeOOH^16^ have all been found to accelerate the rate of Mn(II) oxidation. The catalytic oxidation of Mn(II) by manganese oxides is different from other catalytic oxidation methods. Because of the strong autocatalytic oxidative ability^17^ of active manganese oxides (MnO_*x*_), oxygen oxidizes manganese ions to manganese oxides, and the newly generated manganese oxides also have catalytic oxidation characteristics. The simplified oxidation reaction equations of Mn(II) with dissolved oxygen is as follows^18^:

1 $$2{{\text{Mn}}^{{2 + }}} +{\text{ (x}} -1 {\text{)O}}_{2}  + 2{\text{H}}_{2} {\text{O}}\mathop  \to \limits^{{{\text{MnO}}_{{\text{x}}} }} 2{\text{MnO}}_{{\text{x}}}  + 4{{\text{H}}^{ + }}$$

The formation of active MnO_*x*_ is the key to accelerating the start-up period. The most commonly used method to accelerate the formation of active manganese oxide is to add a strengthening oxidant into RSF system (e.g., ozone, chlorine dioxide, sodium hypochlorite potassium and potassium permanganate)^19,20^. Among these oxidants, potassium permanganate oxidation has obvious advantages. In ordinary RSF, the source of manganese in active MnO_*x*_ is only from groundwater, but adding potassium permanganate brings in extra manganese element into RSF. It is reported that adding potassium permanganate could reduce the start-up period to 36 days^21^. However, adding Potassium permanganate into RSF has certain potential safety hazards, which may cause potassium permanganate in water to exceed the standard. The oxidation reaction equation of Mn(II) with potassium permanganate is as follows:

2 $${{3}{\text{Mn}}}^{2+}\text{+}{2}{\text{Mn}}{\text{O}}_{4}^{-}{+}{2}{\text{H}}_{2}{\text{O}}\stackrel \, {\to }{5}{\text{Mn}}{\text{O}}_{2}\text{+}{4}{\text{H}}^{+}$$

Clinoptilolite is the most common zeolite in nature, which possesses a two-dimensional structure^22^. Clinoptilolite is formed from tetrahedral SiO_4_ and AlO_4_^−^ units, and it contains exchangeable metal cation (e.g., Na^+^, K^+^, Ca^2+^ and Mg^2+^) in its structural framework^23,24^. Clinoptilolite is an ideal material in the field of water treatment due to its adsorption performance, ion exchange ability, and high specific surface area^25,26^. Besides, modified clinoptilolite which is produced through methods such as cation exchange^27^, redox reactions^28^, has a larger surface area and adsorption performance. Obviously, clinoptilolite is more suitable for RSF than quartz sand as filter material. In some regions, the price of natural clinoptilolite is even lower than that of quartz sand.

This study aims to investigate characteristic of manganese oxide film coated clinoptilolite (MOFCC), which combines autocatalytic oxidation ability of manganese oxides with the adsorption ability of clinoptilolite. SEM, EDS, XPS, XRD, ZETA potential and BET analyses were used to observe the surface properties of clinoptilolite and MOFCC. The adsorption capacities of clinoptilolite and MOFCC were examined using the adsorption isotherm technique. Removing Mn(II) from solution using a fixed-bed column for 30 days to verify whether MOFCC has catalytic oxidation characteristics for Mn(II). If the MOFCC in the fixed-bed column can still effectively remove Mn(II) after the cumulative amount of manganese removal exceeds the adsorption capacity, which can be considered that MOFCC has the catalytic oxidation ability for Mn(II).

## Results

### Characteristics of clinoptilolite and MOFCC

**Figure 1:**
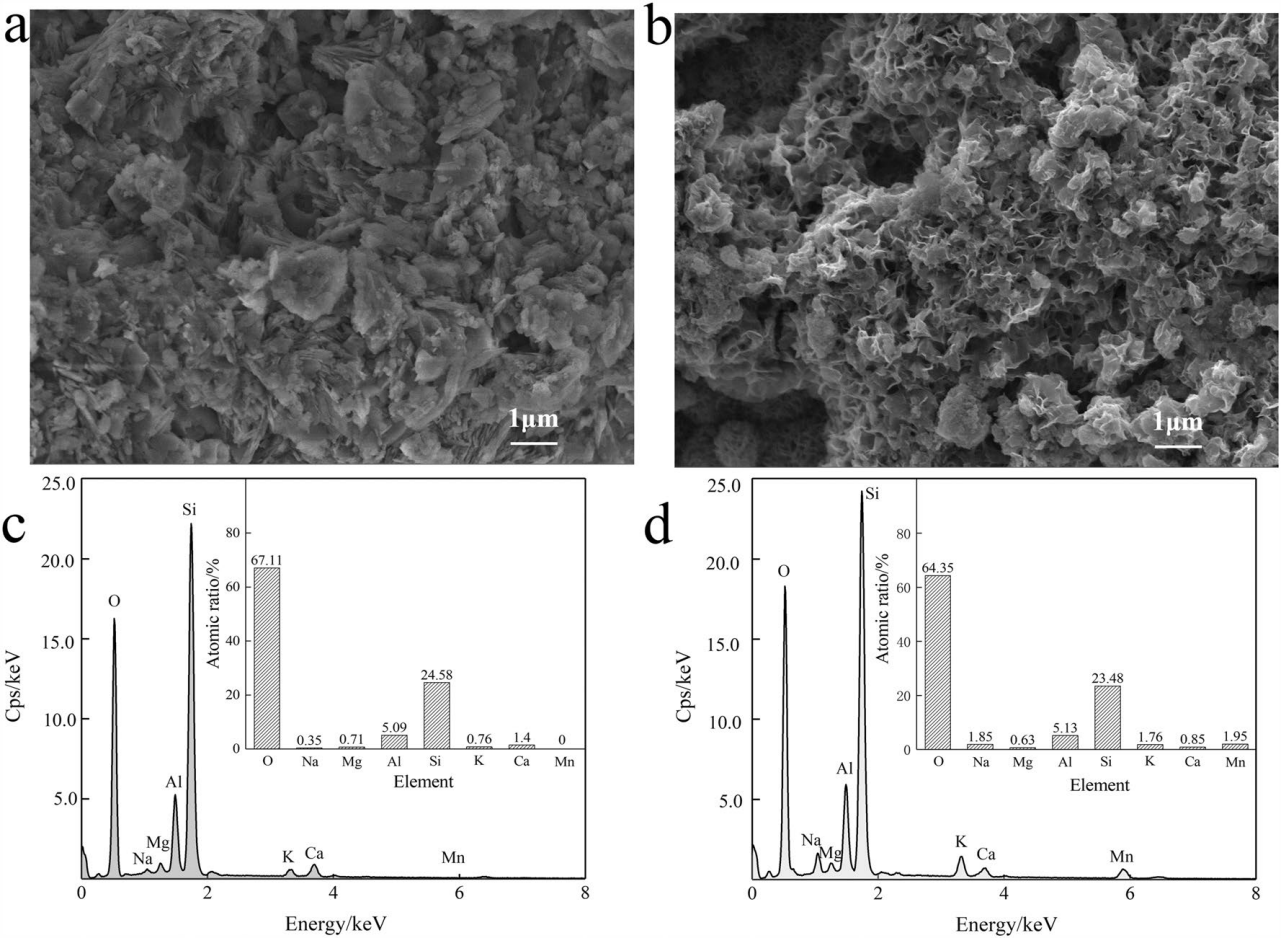
SEM micrograph and EDS mapping spectrum of clinoptilolite and MOFCC. (**a**) SEM micrograph of clinoptilolite. (**b**) SEM micrograph of MOFCC. (**c**) EDS mapping spectrum of clinoptilolite. (**d**) EDS mapping spectrum of MOFCC.

The SEM photographs of clinoptilolite and MOFCC were taken at 20.0 k× magnification to analysis the surface morphology. From the SEM micrograph of clinoptilolite (Fig. 1a), it can be observed that the original material has a distinct rhombic structure, and there are obvious grooves and pores between the rhombic structures. The presence of these structures gives clinoptilolite a larger specific surface area. The SEM micrograph of MOFCC (Fig. 1b) shows that, the original rhombic structure disappears, the surface of clinoptilolite is enveloped by generated manganese oxides, the manganese oxide exhibits a honeycomb shaped pore shape, there are a large number of pores on its surface. MOFCC exhibits a complex three-dimensional network structure as a whole. EDS energy spectrum scanning of clinoptilolite (Fig. 1c) shows that, the main constituent elements of clinoptilolite are O, Si, Al, Ca, K, Mg, and Na. Except for O, Si and Al, the urelement of clinoptilolite, Ca accounts for the highest proportion, and Mn is not detected. It can be found that 1.95% of Mn was detected in EDS energy spectrum scanning of MOFCC (Fig. 1d), the proportion of O, Si, Al, Ca and Mg element atoms has decreased which is because of the manganese oxide film coated on the surface of clinoptilolite. The atomic numbers of K and Na increased from 0.76% and 0.35% to 1.76% and 1.85%, respectively. The increase in atomic numbers of these two elements is due to the use of potassium permanganate and sodium hydroxide in the preparation of MOFCC, which partially adsorbs K^+^ and Na^+^ on the surface of clinoptilolite and manganese oxide film.

**Figure 2:**
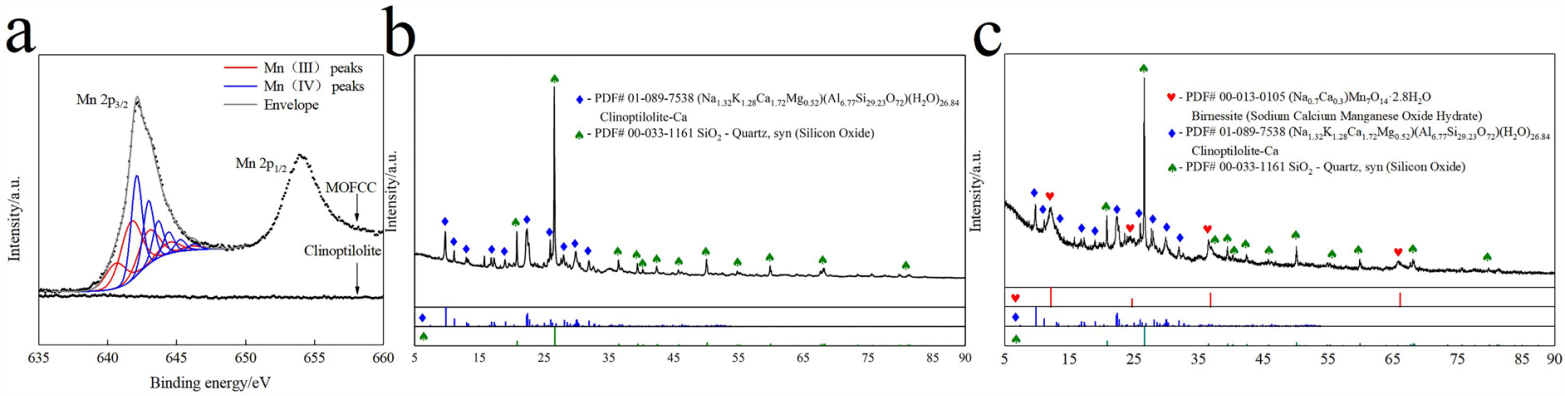
XPS spectra and XRD patterns of clinoptilolite and MOFCC. (**a**) XPS spectra of clinoptilolite and MOFCC. (**b**) XRD patterns of clinoptilolite. (**c**) XRD patterns of MOFCC.

To analyze the valence of manganese in clinoptilolite and MOFCC, XPS analyses were performed, the results are presented in Fig. 2a. In the XPS scanning results of clinoptilolite, there is no obvious peak shape and the intensity is very low, this indicates that clinoptilolite does not contain Mn element. This result is consistent with the EDS analysis results. The XPS scanning results of MOFCC has two obvious peaks, Mn 2p_1/2_ and Mn 2p_3/2_. The element Mn has six stable oxidation state (0, II, III, IV, V, VI, VII). Because Mn(II, III, IV) contains unpaired electrons, there are significant multiplet spiltting in the XPS spectra^29^. Fitting parameters for Mn 2p_3/2_ and the resluts of fitting XPS spectra of MOFCC are presented in Table 1. There is no obvious Mn(II) specific shake-up peak at 645.94 eV, and there are no peaks were obserbved at 638.60 and 645.50 eV, this indicates that MOFCC does not contain Mn(0, II, VII). The fitting results indicate that the existing forms and molar ratios of Mn in MOFCC are 51.28% for Mn(III) and 48.72% for Mn(IV).

**Table 1:** Fitting parameters and content of Mn 2p_3/2_ orbital peaks on the surface of MOFCC.

Compound	Peaks	Binding energy/eV	FWHM/eV	Molar ratio/%	
Mn(0)	Peak 1	638.60	0.74	0.00	0.00
Mn(II)	Peak 1	640.20	1.21	0.00	0.00
	Peak 2	641.17	1.21	0.00	
	Peak 3	642.10	1.21	0.00	
	Peak 4	643.05	1.21	0.00	
	Peak 5	644.19	1.21	0.00	
	Peak 6(shake-up)	645.94	3.50	0.00	
Mn(III)	Peak 1	640.60	1.75	9.65	51.28
	Peak 2	641.70	1.75	22.74	
	Peak 3	642.97	1.75	12.94	
	Peak 4	644.47	1.75	4.36	
	Peak 5	646.09	1.75	1.59	
Mn(IV)	Peak 1	641.90	0.91	20.26	48.72
	Peak 2	642.70	0.91	12.88	
	Peak 3	643.40	0.91	7.54	
	Peak 4	644.20	0.91	4.43	
	Peak 5	645.00	0.91	2.39	
	Peak 6	646.00	0.91	1.22	
Mn(VI)	Peak 1	643.80	1.31	0.00	0.00
Mn(VII)	Peak 1	645.50	0.98	0.00	0.00

^a^FWHM is the half peak width;

^b^The binding energy and FWHM of the multi split peaks were from Handbook of X-ray photoelectron spectroscopy^29^ and references^31^.

**Figure 3:**
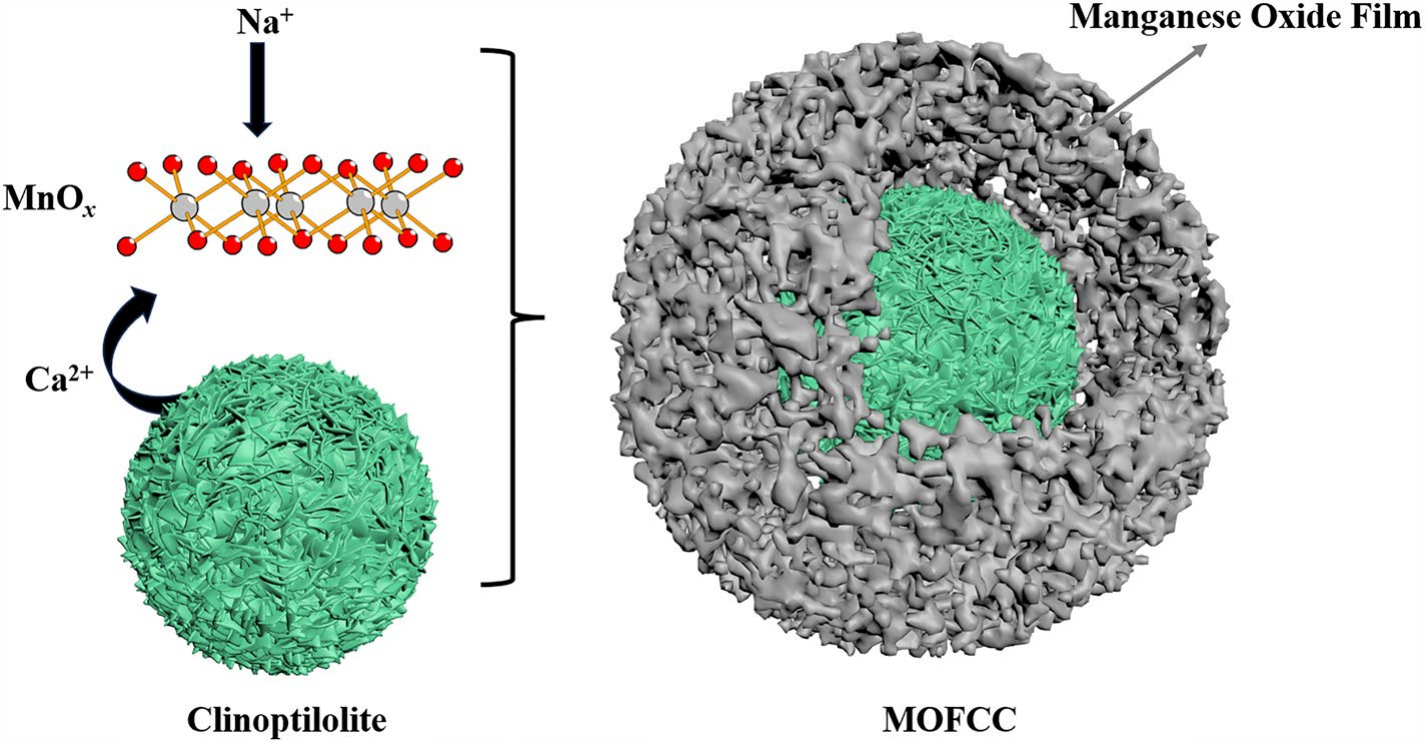
Schematic diagram of the formation process of MOFCC.

The XRD patterns of clinoptilolite and MOFCC were presented in Fig. 2b,c. As shown in Fig. 2b, narrow and intense peaks at 2*θ* = 20.850°, 26.652°, 36.542°, 39.456°, 40.283°, 42.465°, 45.809°, 50.611°, 55.328°,68.322°, 81.471°, that were assigned to SiO_2_ (PDF#00-033-1161, SiO_2_, syn), there are also peaks at 2*θ* = 9.483°, 11.159°, 13.320°, 17.488°, 22.749°, 25.314°, 26.270°, 28.116°, 30.065°, that were assigned to (Na_1.32_K_1.28_Ca_1.72_Mg_0.52_)(Al_6.77_Si_29.23_O_72_)(H_2_O)_26.84_ (PDF#01-089-7538, Clinoptilolite-Ca). This indicates that the clinoptilolite used in this study is composed of quartz sand and calcium type clinoptilolite. In the Fig. 2c, in addition to possessing XRD spectra of clinoptilolite, MOFCC has detected peaks at 2*θ* = 12.164°, 24.710°, 36.806°, 66.122°, that were assigned to (Na_0.7_Ca_0.3_)Mn_7_O_14_·2.8H_2_O (PDF#00-033-1161, Birnessite, Sodium Calcium Manganese Oxide Hydrate). The XRD spectrum analysis results indicate that the manganese oxide film coated on the surface of clinoptilolite is birnessite. Birnessite catalytic oxidation effect has been reported multiple times^30^. It is note worthy that both Ca and Na were involved in the formation of manganese oxide film. The main source of sodium is sodium hydroxide used for pH adjustment. Due to the ion exchange interaction of clinoptilolite, calcium ions in clinoptilolite are exchanged into the solution. Under MnO_*x*_ adsorption effect, MnO_*x*_, Na, Ca and H_2_O, the four of them together formed birnessite, and it coated on the clinoptilolite. The schematic diagram of the formation process is shown in Fig. 3.

**Figure 4:**
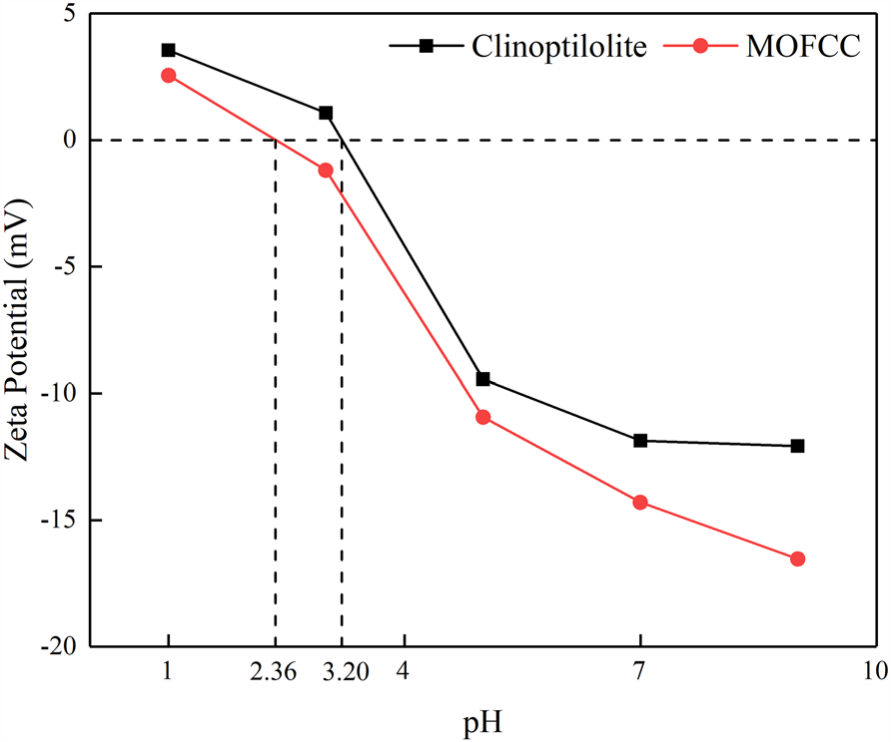
Zeta potential curves of clinoptilolite and MOFCC.

The Zeta potential curves of clinoptilolite and MOFCC are shown in Fig. 4. The change in pH value will affect the hydrolysis equilibrium of the material surface and affect the charged characteristics of the material surface. At pH values ranging from 1 to 9, the zeta potential measurement of clinoptilolite ranges from 3.55 to − 12.07 mV, and the zeta potential measurement value of MOFCC ranges from 2.55 to − 16.53 mV. The zeta potential values of clinoptilolite and MOFCC decrease with the increase of pH value. The point of zero charge of clinoptilolite and MOFCC are near pH 3.20 and pH 2.36. Based on the XRD patterns characterization analysis results, the main component of the manganese oxide film on the surface of MOFCC is sodium calcium manganese oxide hydrate, the point of zero charge is about pH 2.0^32^. Under the coating effect of manganese oxide, the point of zero charge of MOFCC has overall negative shift. When the pH value is less than the point of zero charge, the material surface is positive and has adsorption for anions; when the pH value is greater than the point of zero charge, the material surface is negative and has adsorption for cations. The point of zero charge of MOFCC is lower than that of clinoptilolite, which enables MOFCC to have a wider pH range than clinoptilolite when adsorbing cations. At the same pH value, the surface zeta potential of MOFCC is lower than that of clinoptilolite, indicating that MOFCC has stronger adsorption capacity for cations.

**Figure 5:**
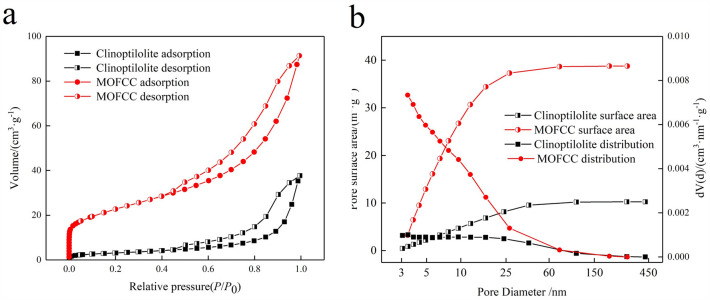
Adsorption and desorption curves, specific surface area and pore size distribution of clinoptilolite s and MOFCC. (**a**) Adsorption and desorption curve. (**b**) Specific surface area and pore size distribution.

The pore volume of MOFCC reaches 91.31 cm^3^ g^−1^, which is much larger than that of clinoptilolite 37.71 cm^3^ g^−1^ (Fig. 5a). When the relative pressure is low, the nitrogen adsorption capacity of clinoptilolite does not increase significantly, belonging to single-layer adsorption. As the relative pressure increases, multi-layer adsorption gradually forms, and the inflection point appears near the saturated vapor pressure (P/P0 = 1). The adsorption capacity of MOFCC increases significantly in the low relative pressure region, indicating that in the small pore size range, MOFCC has more holes. As the relative pressure increases, the curve of MOFCC converges with that of clinoptilolite, indicating that nitrogen diffuses into the interior of MOFCC. Within the large pore size range, the clinoptilolite in the inner layer of MOFCC plays an adsorption role. The specific surface area of clinoptilolite is 10.24 m^2^ g^−1^, and the specific surface area of MOFCC is 38.76 m^2^ g^−1^ (Fig. 5b), the coating of manganese oxide film significantly improves the specific surface area of the material. The pore size of clinoptilolite and MOFCC is concentrated in the range of 3–40 nm, belonging to the mesoporous range. Within the pore size range of 3–10 nm, the number of mesopores in MOFCC is significantly greater than that of clinoptilolite. The increase in mesopores results in a larger specific surface area and more adsorption sites for MOFCC, which is beneficial for improving adsorption capacity.

### Adsorption isotherm equations of clinoptilolite and MOFCC

**Figure 6:**
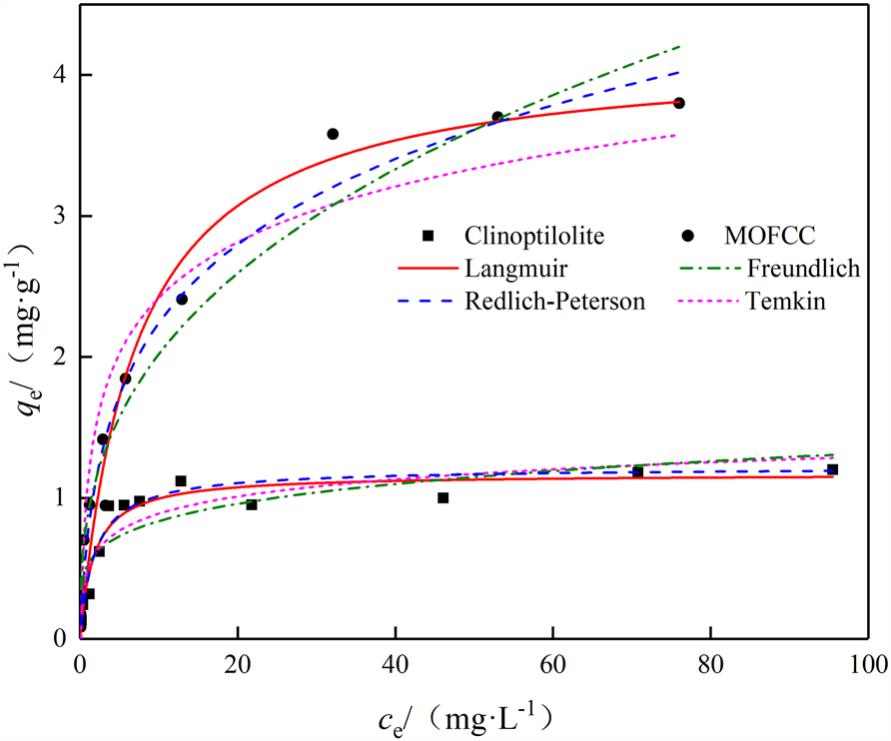
Isothermal equation for adsorption of Mn(II) on clinoptilolite and MOFCC.

The isothermal equation for adsorption of Mn(II) on clinoptilolite and MOFCC is shown in Fig. 6. The equilibrium adsorption capacity of clinoptilolite and MOFCC for Mn(II) increases with the increase of Mn(II) mass concentration. After the mass concentration of Mn(II) exceeds 8 mg L^−1^, the equilibrium adsorption capacity of clinoptilolite increases slowly, and there is almost no change when the concentration of Mn(II) increases to 32 mg L^−1^. Both adsorption isotherms for Mn(II) belong to type I adsorption curves. At the same Mn(II) equilibrium mass concentration, the Mn(II) adsorption capacity of MOFCC is much higher than that of clinoptilolite. This is due to the loading of manganese oxide, which greatly increases the specific surface area and adsorption sites of clinoptilolite. The Langmuir, Freundlich, Temkin and Redlich-Peterson isothermal adsorption models were used to fit and analyze the adsorption process of Mn(II) on natural clinoptilolite and MOFCC. The results are shown in Table 2. The fitting of Langmuir adsorption isotherm for the adsorption process of Mn(II) on clinoptilolite and MOFCC is the best of the four isotherms. The *R*^2^ are 0.934 and 0.970, respectively for clinoptilolite and MOFCC, the *q*_m_ are 1.17 and 4.17 mg g^−1^.

**Table 2:** Isothermal parameters for adsorption of clinoptilolite and MOFCC.

Isothermal model parameters	Parameters	Clinoptilolite	MOFCC
Langmuir	*K*_L_	0.57	0.14
	*q*_m_	1.17	4.17
	*R*^2^	0.934	0.970
	Equation	*q*_e_ = 0.669*C*_e_/(1 + 0.57 *C*_e_)	*q*_e_ = 0.584* C*_e_/(1 + 0.14* C*_e_)
Freundlich	*K*_F_	0.53	0.88
	n	5.06	2.77
	*R*^2^	0.754	0.962
	Equation	*q*_e_ = 0.53*C*_e_^(1/5.06)^	*q*_e_ = 0.88*C*_e_^(1/2.77)^
Temkin	*A*	0.49	1.10
	*B*	0.18	0.57
	*R*^2^	0.859	0.924
	Equation	*q*_e_ = 0.49 + 0.18ln*C*_e_	*q*_e_ = 1.10 + 0.57ln*C*_e_
Redlich-Peterson	*K*_RP_	0.66	1.39
	*α*_RP_	0.55	0.87
	g	0.995	0.78
	*R*^2^	0.929	0.968
	Equation	*q*_e_ = 0.66*C*_e_/(1 + 0.55*C*_e_^0.995^)	*q*_e_ = 1.39*C*_e_/(1 + 0.87* C*_e_^0.78^)

### Manganese removal from fixed-bed columns

**Figure 7:**
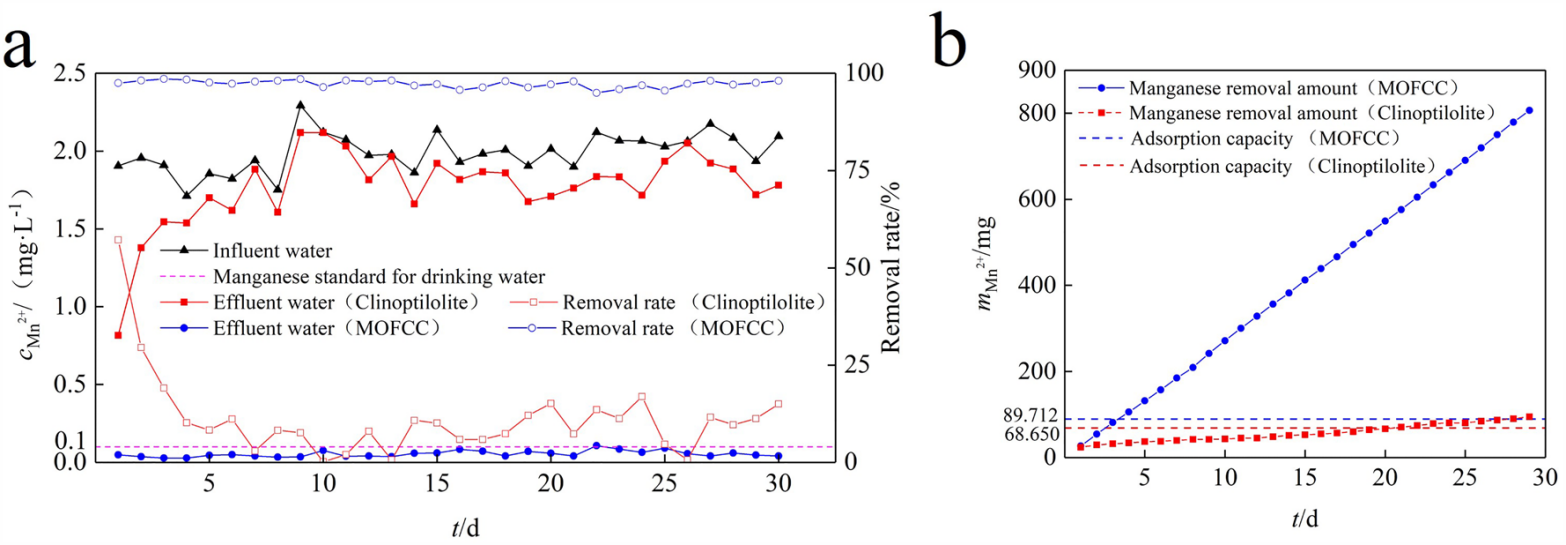
Continuous Mn(II) removal curves and accumulated manganese removal by clinoptilolite and MOFCC fixed-bed columns. (**a**) Continuous Mn(II) removal. (**b**) Accumulated manganese removal.

The comparison of manganese removal efficiency in fixed-bed columns between clinoptilolite and MOFCC through continuous filtration is shown in Fig. 7a. Clinoptilolite has a certain manganese removal ability in the early stage of filtration. On the first day, the mass concentration of Mn(II) in the filtered water was 0.82 mg L^−1^, with a removal rate of 57.26%. As the operating time prolongs, the mass concentration of Mn(II) in the filtered water gradually increases and the removal rate decreases. On the 5th day, the ability to remove manganese was basically lost, and the removal rate decreased to around 10%. During the 30 days operation cycle of Mn(II) removal from MOFCC fixed-bed columns, the manganese removal effect is much better than clinoptilolite, and the removal rate has been maintained at over 95%. The mass concentration of Mn(II) in the filtered water is always below 0.1 mg L^−1^, which meets the requirements of the hygienic standards for drinking water in China. The cumulative Mn(II) removal amount of clinoptilolite fixed-bed column 94.74 mg, and that of MOFCC is 806.42 mg (Fig. 7b). According to the Langmuir model calculation of clinoptilolite and MOFCC, the adsorption capacity of the filter columns is 68.650 and 89.712 mg, respectively. It can be seen that the cumulative Mn(II) removal amount of clinoptilolite fixed-bed column is close to the adsorption capacity, while the cumulative Mn(II) removal amount of MOFCC far exceeds the adsorption capacity, indicating that the removal of Mn(II) from water by MOFCC is not only due to adsorption effect, but also from catalytic oxidation process. It is reported that, manganese oxide can effectively reduce the concentration of Mn(II) in water^18^. The removal process involves the adsorption of Mn(II) on the surface of manganese oxides. Under the catalytic action of manganese oxides, Mn(II) is oxidized by dissolved oxygen to a higher valence manganese oxide, which precipitates on the surface of the oxide. The newly generated manganese oxide continues to adsorb and catalyze the oxidation of Mn(II), forming an autocatalyzed oxidation process. This mechanism of action is also the mechanism of manganese removal in the water purification plant RSF process. However, the start-up period in RSF takes a long time. But, during the MOFCC fixed-bed column Mn(II) removal process, after exceeding the adsorption capacity, the removal rate remains above 95%, indicating that MOFCC does not require a start-up period for manganese removal. MOFCC is a good filter material that can be applied in the RSF process of water treatment plants.

## Discussions

In this study, the structural characteristics, chemical composition, adsorption characteristics and catalytic oxidation characteristics of MOFCC which prepared by loading manganese oxides generated by potassium permanganate and Mn(II) sulfate on the surface of clinoptilolite, were analyzed. The manganese oxide film alters the surface structure and chemical composition of clinoptilolite presenting a complex three-dimensional network structure. The existing forms and molar ratios of Mn elements are 51.28% for Mn(III) and 48.72% for Mn(IV), respectively, the compound formed is (Na_0.7_Ca_0.3_)Mn_7_O_14_·2.8H_2_O, calcium ion in clinoptilolite and sodium ion in sodium hydroxide participate in the formation of manganese oxide film. The surface area of MOFCC is 38.76 m^2^ g^−1^, the pore diameter is concentrated at 3–40 nm, and the isoelectric point pH is 2.36. The point of zero charge of MOFCC are near pH 2.36. The Langmuir adsorption isotherm equation can well describe the process of manganese ion adsorption by MOFCC. MOFCC has the ability to autocatalytic oxidation of manganese ions, does not require the start-up period, it is a good filter material that can be applied in RSF of water treatment plants.

## Methods

### Preparation of MOFCC

The raw clinoptilolite used in this research obtained from Huludao city in China. The clinoptilolite were crushed and sieved through 20 mesh sieve, rinsed with distilled water, and dried at 378 K. A solution containing potassium permanganate was poured over dried clinoptilolite in a beaker, then respectively, dropwise, add sodium hydroxide and Mn(II) sulfate solution, maintain the pH of the solution at around 7.0, until the solution turns colorless. The media was filtered, dried at 378 K, manganese oxide film was coated on the surface of clinoptilolite. Store the MOFCC in a polypropylene bottle for use.

### Characterization of MOFCC and clinoptilolite

The characteristics of MOFCC and clinoptilolite surfaces were explored by scanning electron microscopy (SEM, TESCAN MIRA LMS, CZ). The element distribution was analyzed using the mapping analysis of energy dispersive spectroscopy (EDS, TESCAN, Xplore 30, CZ), and the element valences were analyzed by curve fitting the X-ray photoelectron spectroscopy (XPS, Thermos Scientific K-Alpha, USA). X-ray diffraction (XRD, SmartLab-SE) were using to analyze the structure of MOFCC and clinoptilolite. Zeta potential and the point of zero charge (pH_pzc_) were measured to analyze surface charge (Nano-ZS ZEN3600, UK). The BET adsorption model was used to calculation the specific surface area, and the BJH model were used to calculate the size of pore (Autosorb-IQ-MP, USA).

### Adsorption isotherm of MOFCC and clinoptilolite

The oxides of Si(IV), Mn(III), Mn(IV)^33^ have been found that they can accelerate the oxidation of Mn(II). In order to accurately measure the adsorption process of manganese ions on clinoptilolite and MOFCC, and reduce the decrease in Mn(II) concentration caused by oxidation, the adsorption isotherm testing is conducted in an anaerobic chamber, under a nitrogen atmosphere. In order to be close to the actual environmental conditions of water treatment plants, the adsorption experiments were performed at 25 °C, pH 7.0. The pH was adjusted to the required value using HCl and NaOH, Dissolved oxygen meter and pH meter (HACH, HQ-40d, USA) were used to maintain stable adsorption environment. Manganese containing water was prepared with Mn(II) sulfate monohydrate. All solutions in this study were prepared with deionized water, and all chemicals and reagents were of analytical grade. The solutions were filtered through 0.45-μm filters. Filterable manganese was determined the form aldoxime method^34^ (HACH, DR-3900, USA). The amount of adsorbed of Mn(II) per gram MOFCC was obtained using the following expression:

3 $${\text{Q}}_{\text{e}}\text{=}\left({\text{C}}_{0}-{\text{C}}_{\text{e}}\right){V}_{0}\text{/}{\text{m}}$$

where *Q*_e_ is the amount of Mn(II) adsorbed on the MOFCC or clinoptilolite, *C*_0_ and *C*_e_ are the concentrations of Mn(II) in the solution (mg L^−1^) prior to and after adsorption, *V*_0_ is the solution volume, *m* is the weight of the MOFCC or clinoptilolite.

The Langmuir^35^, Freundlich^36^, Temkin^37^, Redlich-Peterson^38^ adsorption isotherm equations were used to analysis the process of MOFCC and clinoptilolite adsorption Mn(II).

4 $${\text{Langmuir: }{\text{{C}}_{{e}}{/}{{q}}}_{\text{e}}\text{=1/(}{\text{K}}_{{L}}{{{q}}}_{{m}}{)+}{{C}}_{{e}}{/}{{q}}_{{m}}}$$

5 $$\text{Freundlich: }{{\text{q}}_{{e}}{=}{{K}}_{{F}}{{{C}}}_{\text{e}}^{{1/n}}}$$

6 $$\text{Temkin: }{{\text{q}}_{{e}}\text{=}{RT }{ln(}{\alpha }_{t}{{C}}_{{e}}{)/}{b}_{t}{=}{{A}}{+}{{B}}{{ln}}{{C}}_{{e}}}$$

7 $$\text{Redlich-Pet}{\text{erson: }{{q}}_{{e}}\text{=}{{K}}_{{RP}}{{{C}}}_{{e}}{/(1+}{\alpha }_{{RP}}{{{C}}}_{{e}}^{{g}}{)}}$$

where *C*_e_ (mg L^−1^) is the equilibrium Mn(II) concentration, *K*_L_ (L mg^−1^) is the Langmuir equilibrium adsorption constant, *q*_m_ (mg g^−1^) is the maximum amount of Mn(II) ion per unit weight of clinoptilolite and MOFCC, *K*_F_ (mg g^−1^) and *n* is the Freundlich equilibrium adsorption constant, *R* (J mol^−1^ K^−1^) is the general gas constant, *T* (K) is absolute temperature, *α*_t_, *b*_t_,* A* [= (*RT*ln*α*_t_)/*b*_t_] and *B* (= RT/*b*_t_) are Temkin isotherm constants, *K*_RP_, *α*_RP_ and g are Redlich-Peterson isotherm constants.

### Mn(II) removal in fixed-bed columns

**Figure 8:**
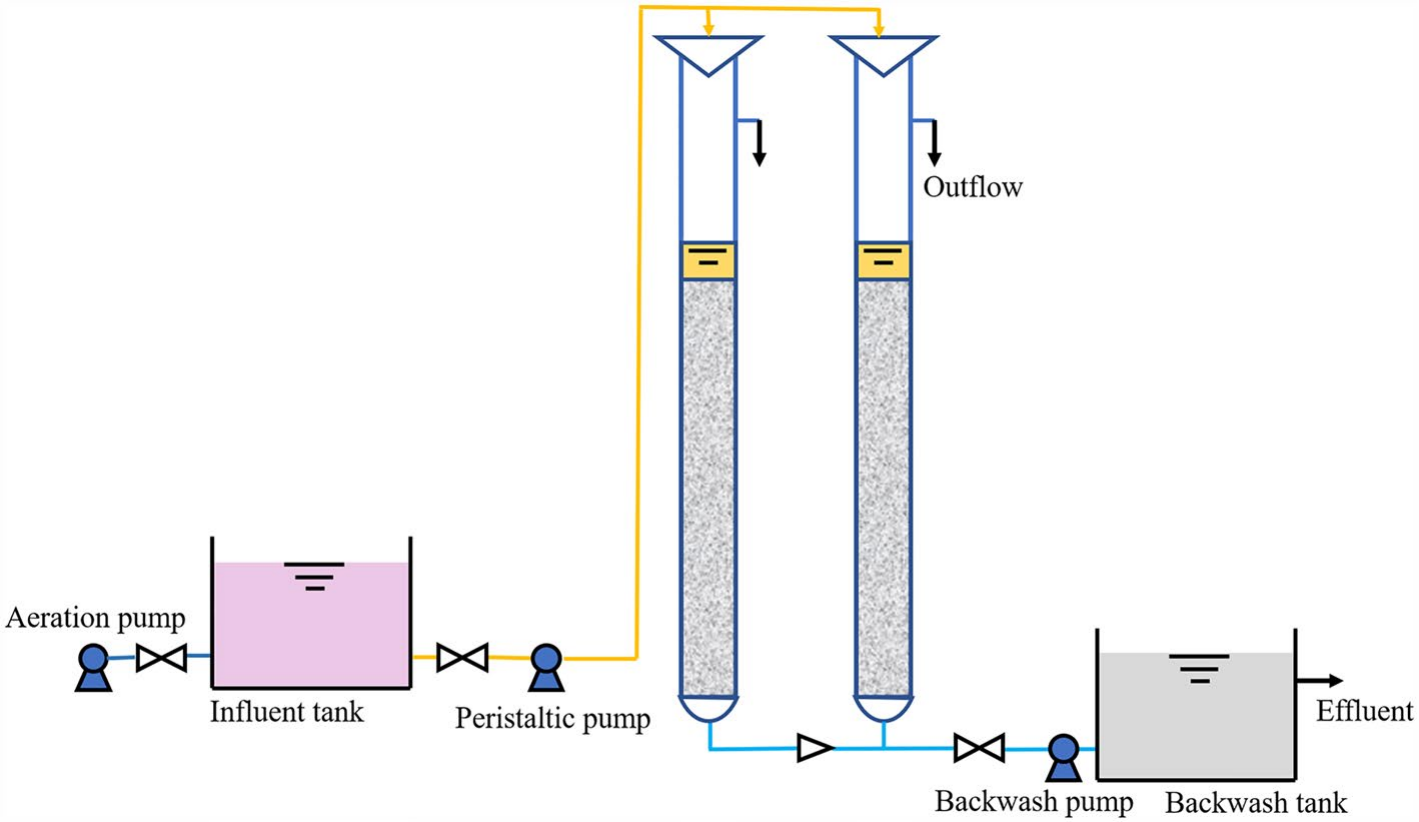
Fixed-bed columns Mn(II) removal system used in this research.

Mn(II) sulfate monohydrate is used to prepare synthetic wastewater, the concentration of Mn(II) in the influent is 2 mg L^−1^, and Mn(II) removal experiments were conducted in clinoptilolite and MOFCC fixed-beds columns. The setup of the system is displayed in Fig. 8. Two identical plexiglass cylinders were used in the system, each plexiglass cylinders were with an inner diameter of 80 mm, and a height of 300 mm. Filter layer height was 80 mm, filter layer volume was 100 cm^3^. In the fixed-bed columns of clinoptilolite, the mass of filter material is 110.0 g, and in the fixed-bed columns of MOFCC, the mass was 80.1 g. The hydraulic retention time (HRT) was 10 min, physical backwashing was conducted every 12 h, at the water flow in tensity of 5 L/(m^2^ s^−1^), for 5 min. The fixed-bed columns Mn(II) removal experiments was performed for 30 days. Calculate the adsorption capacity of a fixed-bed columns based on the adsorption isotherm equation of clinoptilolite and MOFCC, using the following formula:

8 $${\text{m}}_{\text{a}}={\text{q}}_{\text{e}}{{\text{m}}}_{0}$$

where *m*_a_ (mg) is the adsorption capacity of the fixed-bed columns, *m*_0_ (g) is the mass of the clinoptilolite or MOFCC.

Calculate the cumulative manganese removal amount based on the daily mass concentration of Mn(II) in the influent, effluent and the volume of filtered water. When the cumulative manganese removal amount exceeds the adsorption capacity of the fixed-bed columns, the filter material can still continuously remove Mn(II), it can be considered that the filter material has already possessed the ability of autocatalyzed oxidation. The formula is as

9 $${\text{m}}=\sum_{0}^{\text{n}}({C}_{\mathrm{I}}-{C}_{\mathrm{E}}){\text{V}}_{\text{n}}$$

where *m* (mg) is the cumulative manganese removal amount, n (d) is the number of days the system is running, *C*_I_ and *C*_E_ (mg L^−1^) are the concentration of Mn(II) in the influent and effluent, *V*_n_ (L) is the volume of filtered water on day n.

### Supplementary Information

Supplementary Information.

## Data Availability

All data generated or analysed during this study are included in this published article and its supplementary information files.
